# The interferon-induced protein, IFIT2, requires RNA-binding activity and neuronal expression to protect mice from intranasal vesicular stomatitis virus infection

**DOI:** 10.1128/mbio.00568-24

**Published:** 2024-06-18

**Authors:** Darshana Poddar, Nikhil Sharma, Tomoaki Ogino, Xu Qi, Patricia M. Kessler, Hiran Mendries, Ranjan Dutta, Ganes C. Sen

**Affiliations:** 1 Department of Inflammation and Immunity, Lerner Research Institute, Cleveland Clinic, Cleveland, Ohio, USA; 2 Department of Medical Microbiology and Immunology, College of Medicine and Life Sciences, The University of Toledo, Toledo, Ohio, USA; 3 Department of Neurosciences, Lerner Research Institute, Cleveland Clinic, Cleveland, Ohio, USA; Columbia University Medical Center, New York, New York, USA

**Keywords:** IFIT2, interferon, neurotropic viruses

## Abstract

The interferon (IFN) system protects mammals from diseases caused by virus infections. IFN synthesis is induced by pattern recognition receptor signaling pathways activated by virus infection. IFN is secreted from the infected cells and acts upon neighboring cells by binding cell surface receptors and triggering induction of hundreds of IFN-stimulated genes and proteins, many of which block different steps of virus replication. The IFN-induced tetratricopeptide repeat proteins (IFIT) are a family of RNA-binding proteins. We and others have previously reported that IFIT2 protects mice from many neurotropic RNA viruses; indeed, Ifit2−/− mice are very susceptible to intranasal or subcutaneous infections with vesicular stomatitis virus (VSV). Here, using a newly generated conditional knockout mouse, we report that ablation of Ifit2 expression only in neuronal cells was sufficient to render mice susceptible to neuropathogenesis caused by intranasal, but not subcutaneous, infection of VSV. Another genetically modified mouse line, expressing a mutant IFIT2 that cannot bind RNA, was as susceptible to VSV infection as *Ifit2*−/− mice. These results demonstrated that IFIT2 RNA-binding activity is essential for protecting mice against neurological diseases caused by intranasal infection of VSV.

**IMPORTANCE:**

Interferon’s (IFN’s) antiviral effects are mediated by the proteins encoded by the interferon-stimulated genes. IFN-stimulated genes (IFIT2) is one such protein, which inhibits replication of many RNA viruses in the mouse brain and the resultant neuropathology. Our study sheds light on how IFIT2 works. By ablating Ifit2 expression only in neuronal cells, using a newly generated conditional knockout mouse line, we showed that Ifit2 induction in the neurons of the infected mouse was necessary for antiviral function of interferon. IFIT2 has no known enzyme activity; instead, it functions by binding to cellular or viral proteins or RNAs. We engineered a new mouse line that expressed a mutant IFIT2 that cannot bind RNA. These mice were very susceptible to infection with vesicular stomatitis virus indicating that the RNA-binding property of IFIT2 was essential for its antiviral function *in vivo*.

## INTRODUCTION

The type I interferon (IFN) system is instrumental in protecting mammals from virus infection. Different viral components, primarily nucleic acids, are recognized by cellular pattern recognition receptors (PRR), which trigger IFN synthesis (1, 2). Newly induced IFN is secreted from the infected cells and protects the neighboring uninfected cells by establishing an antiviral state in them. Interferon-α/β receptor (IFNAR), the cell surface heterotrimeric receptor for all type I IFNs, is ubiquitously expressed. Upon binding type I IFNs, IFNAR activates the Janus kinase/signal transducers and activators of transcription signaling pathway leading to transcriptional activation of more than 200 IFN-stimulated genes (ISG) (3, 4). Although IFN-induced non-coding RNAs may have cellular functions (5), the proteins encoded by ISGs carry out all biological functions of IFN including its antiviral effects. *In vivo*, IFN’s antiviral effects are multifaceted; some ISG products provide cell intrinsic antiviral immunity, whereas others augment the functions of different immune cells to facilitate elimination of the infected cells (6, 7). Consequently, the IFNAR−/− mice are very susceptible to many viral diseases (8, 9). To counteract the antiviral effects of the IFN system, almost all viruses have evolved ways to block IFN induction or IFN action or both. In nature, we observe a dynamic equilibrium between the antiviral effects of the IFN system and the evading armaments of different viruses (10).

The biochemical and antiviral functions of a number of ISGs have been studied in detail revealing several general principles; different ISGs can target viruses of different families, and several ISGs can block different steps of replication of the same virus, thereby producing cumulative antiviral effects (11). Recent studies using ISG knock-out mice have revealed unexpected tissue specificity of their functions (12, 13). We have been studying antiviral and biochemical properties of the IFIT (IFN-induced proteins with tetratricopeptide repeats) proteins. This family of proteins (three to four members) is strongly induced by IFNs and by many PRR signaling pathways that activate the transcription factors IRF3 or IRF7 (14). There are at least three Ifit genes in mouse, Ifit1, Ifit2, and Ifit3, which encode proteins with similar structures but distinct properties. IFIT proteins do not have enzyme activities; instead, their functions are mediated by their ability to bind specific cellular and viral RNAs and proteins (14–16). IFIT proteins can form heteromeric complexes with other members of the family, which can have distinct functions (12, 17, 18).

The functions of IFIT proteins *in vivo* have been investigated using genetically modified mouse lines. The Ifit genes are clustered, and mice missing the complete Ifit locus (19) or only one Ifit gene have been generated (18, 19). We have focused on investigating the *in vivo* antiviral activity of IFIT in mice. Our prior results clearly showed that Ifit2 provides protection against neuropathogenesis caused by many RNA viruses such as vesicular stomatitis virus, rabies virus, mouse hepatitis virus, and West Nile virus (13, 20–22). Intranasal inoculation with a very low dose of VSV caused rapid spread of the virus in the brains of Ifit2−/− mice and their eventual death (20). Surprisingly, other organs of Ifit2−/− mice, but not IFNAR−/− mice, were as well protected from VSV as those of wild-type (Wt) mice, indicating that other ISGs, not Ifit2, are responsible for protection of tissues other than the brain (20). In another model of neuropathogenesis by VSV, the virus was injected into the footpad of mice causing hind leg paralysis, which was much more pronounced in Ifit2−/−mice (23). Thus, Ifit2 protects both the central and peripheral nervous system from viral infections.

In the current study, by engineering a conditional knockout mouse line, we asked whether IFIT2 needs to be expressed in neuronal cells, as against myeloid or other types of cells in the brain, to impart its protective effect. IFIT2, like other IFIT proteins, can bind RNA. To investigate whether its RNA-binding ability is required for antiviral activity *in vivo*, we generated a knock-in mice expressing an Ifit2 mutant that cannot bind RNA. Our results show that both neuronal expression and RNA-binding ability of IFIT2 are required for its protective anti-VSV effects in the brain.

## RESULTS

### *Ifit2* expression in neuronal cells is essential for protecting mice from intranasal infection, but not footpad injection, of VSV

**Fig 1:**
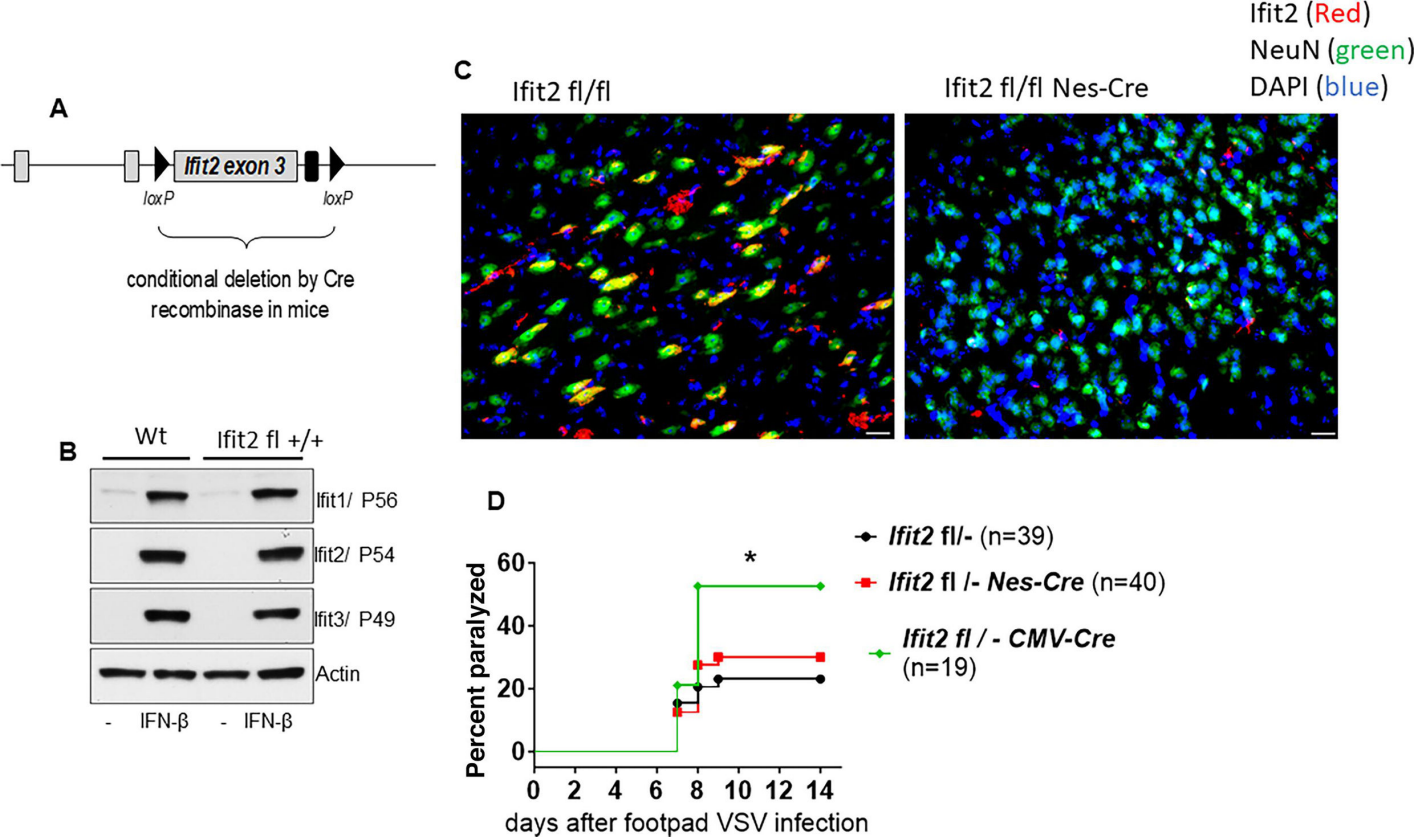
*Ifit2* expression in neuronal cells is not required for preventing paralysis in mice infected with VSV subcutaneously. (**A**) Design of the loxP-flanked (“floxed”) *Ifit2* allele in mice, for conditional, tissue-specific deletion by Cre recombinase. (**B**) IFIT protein expression in splenocytes of Wt and *Ifit2 fl/fl* mice, induced by 8 h of IFN treatment and detected by immunoblot. (**C**) Elimination of IFIT2 induction in neurons of infected *Ifit2 fl/fl*, Nes-Cre mice. Brain sections were stained with anti-IFIT2 (red) to identify IFIT2-expressing cells, anti-NeuN (green) to identify neurons, and 4′,6-diamidino-2-phenylindole (blue) to identify nuclei of all cells. Neurons expressing IFIT2 appear orange in color. (**D**) Kinetics of hind leg paralysis in mice of the indicated genotypes after foot pad injection of VSV. The number of infected mice of each genotype is shown in parenthesis.

We have previously reported that *Ifit2−/*− mice are more susceptible to neurological diseases caused by VSV infection (20). We wanted to identify the cell types that need to express Ifit2 for protecting the infected mice. For this purpose, we generated a new mouse line in which *Ifit2* exon 3, which encodes almost the entire protein, was flanked by two loxP sites (Fig. 1A). By crossing these mice with mice expressing Cre recombinase in specific cell types, we generated various mouse lines that had the *Ifit2* gene deleted only in those cell types. To ensure that the loxP insertion had not altered the induction pattern of Ifit2 and other adjacent Ifit genes, we isolated splenocytes from Wt and *Ifit2 fl+/+* (*Ifit2 fl/fl*) mice and stimulated them *in vitro* with IFN. The three IFIT proteins, IFIT1, IFIT2, and IFIT3, were all strongly induced in cells of both genotypes (Fig. 1B) demonstrating that the Ifit locus of the *Ifit2 fl/fl* mice functioned normally. These mice were crossed with CMV-Cre mice, which express Cre in all cell types, or Nes-Cre mice, which express Cre only in cells of neuroectodermal origin, principally neurons, astrocytes, and oligodendrocytes. Intranasal VSV infection caused IFIT2 induction in neuronal cells in the brains of *Ifit2 fl/fl* mice, but not if they were also expressing Nes-Cre, demonstrating neuron-specific deletion of the *Ifit2* gene in *Ifit2 fl/fl*, Nes-Cre mice (Fig. 1C). When VSV is injected into the footpad of a mouse, the virus traffics to the sciatic nerve and eventually to the brain, causing hind leg paralysis. We used this model to test VSV susceptibility of the newly generated *Ifit2 fl/fl* mice. For these experiments, we used *Ifit2 fl/*− mice, the genome of which had only one allele of the *Ifit2 fl* gene and a null allele. They were crossed with CMV-Cre to delete the *Ifit2 fl* allele in all cell types or with Nes-Cre to delete it in only neuronal cells. The CMV-Cre-expressing mice were quite susceptible to paralysis upon footpad injection of VSV, but the Nes-Cre-expressing mice were as resistant as the *Ifit2 fl/*− mice (Fig. 1D). This observation indicates that *Ifit2* expression in cells, other than neurons, provides protection in this model of pathogenesis.

**Fig 2:**
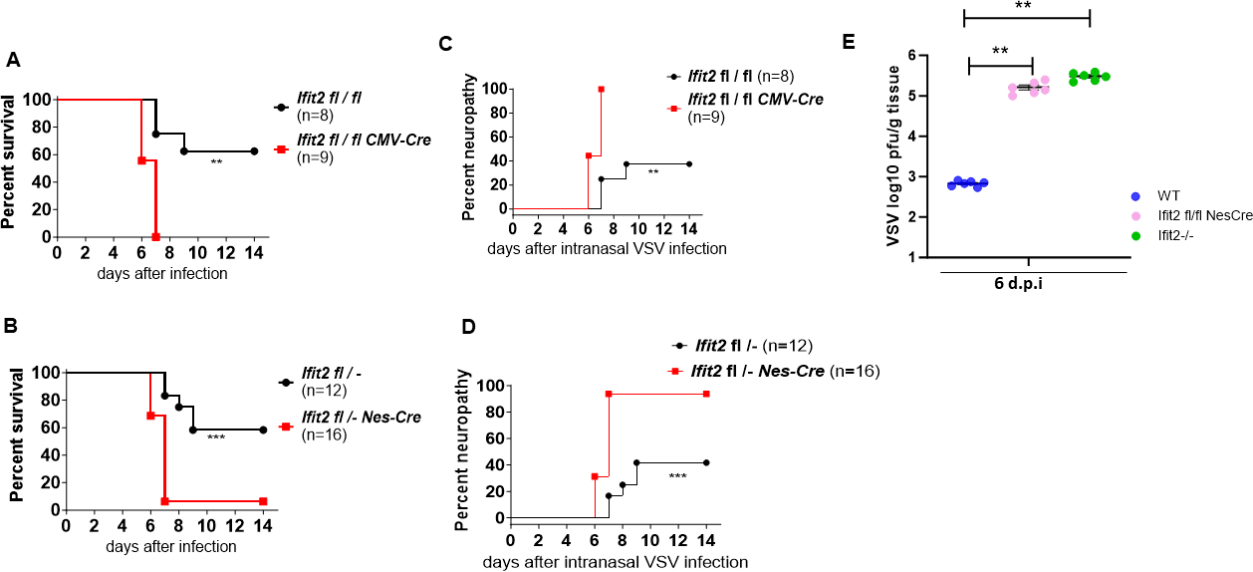
Expression of *Ifit2* in neuronal cells is needed for protecting mice from neuropathy and death caused by intranasal VSV infection. (**A**) Survival of mice with global *Ifit2* deletion (*Ifit2 fl/fl* CMV Cre) or no deletion (*Ifit2* fl/fl) after intranasal VSV infection. CMV-Cre mice ubiquitously express Cre via CMV promoter. (**B**) Survival of mice with conditional Ifit2 deletion in brain cells (neurons, astrocytes, and oligodendrocytes, *Ifit2 fl/*− Nes-Cre mice) after intranasal VSV infection. (**C, D**) Neurological symptoms in mice of the indicated genotypes after intranasal infection with VSV. (**E**) Virus titers in the brains of mice of the indicated genotypes 6 days post infection.

In contrast to the footpad infection model, when mice were infected intranasally with VSV, *Ifit2* expression in neuronal cells was essential to prevent death (Fig. 2A and B) and neurological diseases (Fig. 2C and D). Neither CMV-Cre *Ifit2 fl/fl* mice (Fig. 2A) nor Nes-Cre *Ifit2 fl/*− mice (Fig. 2B) survived beyond 6 days after intranasal infection with a low dose [400 plaque-forming units (pfu)] of VSV, whereas 60% of *Ifit2 fl/fl* mice, without Cre, survived beyond 14 days post infection. Similarly, almost 100% of CMV-Cre *Ifit2 fl/fl* and Nes-Cre *Ifit2 fl/*− mice exhibited various neuropathic symptoms after infection, whereas only approximately 40% of *Ifit2 fl/fl* mice, without Cre, displayed such symptoms (Fig. 2C and D). The morbidity and mortality of the infected Nes-Cre *Ifit2 fl/fl* mice were accompanied by virus replication in the brain to a much higher titer, similar to that in *Ifit2−/*− mice, compared to the titer in Wt mice (Fig. 2E). These results demonstrated that direct infection of the brain by the intranasal route is fatal to mice, unless *Ifit2* can be induced in their neurons in which VSV primarily replicates (24).

### The RNA-binding ability of IFIT2 is required for its protective effects against intranasal VSV infection

**Fig 3:**
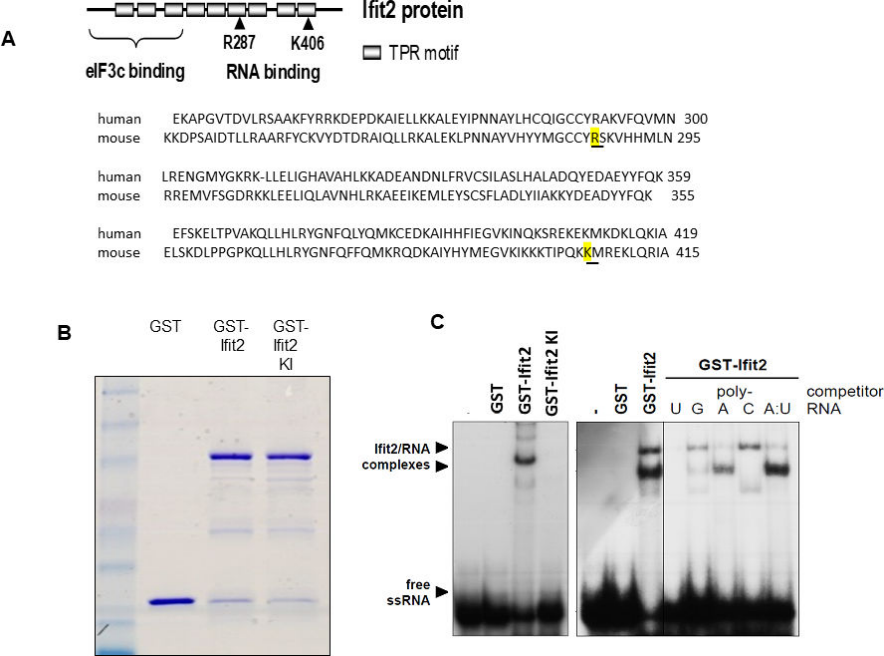
The KI mutant of IFIT2 cannot bind RNA. (**A**) Design of the RNA binding-deficient IFIT2 mutant “KI” (R287E/K406E) (**B**) Detection of purified bacterially expressed GST-fused IFIT2 and IFIT2-KI by Coomassie Blue staining. (**C**) Electrophoretic mobility shift of 32-nucleotide AU-rich single-stranded RNA by IFIT2 binding and competition of the binding by various polynucleotides.

We anticipated that IFIT2, like other IFITs, can bind RNA. Human IFIT2 is known to bind AU-rich regions of RNA, which requires the presence of a specific Arg and a specific Lys residue in that protein (25). Although human and mouse IFIT2 have distinct sequences (Fig. 3A), we speculated that the residues in mouse IFIT2, that are critical for RNA-binding, are Arg 287 and Lys 405. To test it experimentally, we mutated these two residues to Glu. The Wt and mutant (KI) proteins were expressed in *Escherichia coli* as glutathione S-transferase (GST) fusion proteins and purified by affinity chromatography (Fig. 3B). The RNA-binding abilities of the two proteins were tested by electrophoretic mobility shift assays using a short AU-rich RNA probe. Wt murine IFIT2 bound to the probe giving rise to at least two complexes, whereas the KI mutant of IFIT2 or GST alone failed to bind RNA (Fig. 3C, left panel) confirming that R287 and K406 of IFIT2 are required for RNA binding. In another experiment, different polynucleotides were tested for their ability to compete for binding to Wt IFIT2. Formation of the two complexes was differently inhibited by different competitors; 50× excess of poly U competed out both complexes completely, whereas poly A eliminated the slower complex, and poly C eliminated the faster complex (Fig. 3C, right panel). These results suggest that murine IFIT2 can form different RNA complexes by preferential recognition of different nucleotide bases.

**Fig 4:**
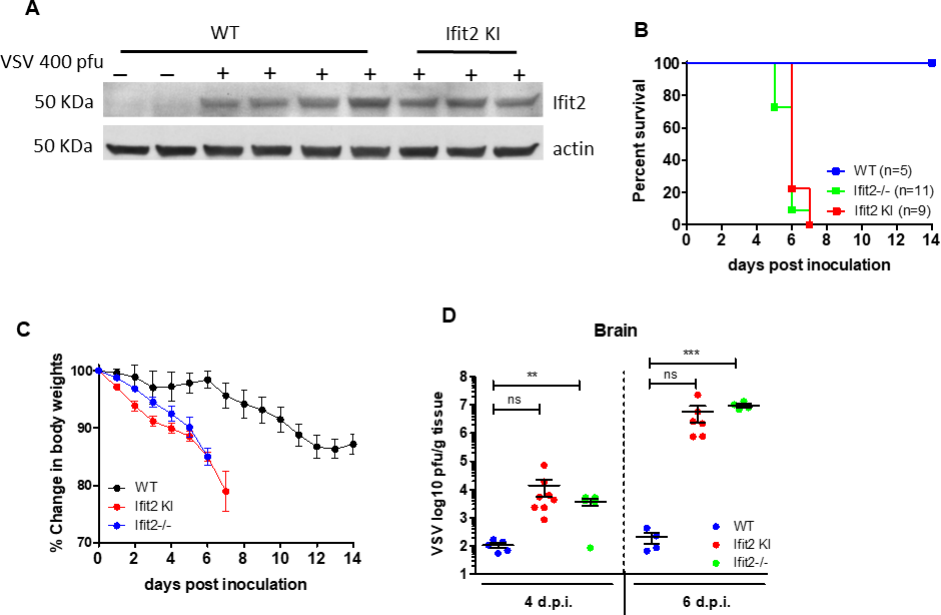
The IFIT2 KI mutant does not protect mice against intranasal VSV infection. (**A**) Mutant IFIT2 is induced at normal levels in the brain, after intranasal VSV infection of the knock-in (KI) mouse. Whole brains of two uninfected, four infected Wt mice, and three infected KI mice were harvested after 3 days of infection and analyzed for IFIT2 expression by immunoblotting. Wt, *Ifit2*−/−, and *Ifit2* KI mice were compared after intranasal VSV infection for (**B**) survival, (**C**) weight loss, and (**D**) virus replication.

A knock-in mutant mouse, expressing the RNA-binding deficient IFIT2, was engineered by introducing the two mutations to the endogenous Ifit2 gene. To ensure that the mutant protein is expressed normally and not unstable, we measured the levels of IFIT2 in the brains of several Wt and KI mice after VSV infection. IFIT2 was induced strongly, and to similar levels in the brain, after intranasal VSV infection (Fig. 4A). The KI mice, along with Wt and Ifit2−/− mice, were challenged with intranasal VSV infection, and the pathogenic consequences in mice of the three genotypes were compared. None of KI and *Ifit2−/*− mice survived beyond day 7 post infection, whereas all Wt mice survived in this experiment (Fig. 4B). Moreover, the KI mice lost weight at a similar rate compared to *Ifit2−/*− mice, whereas the rate was much slower for Wt mice (Fig. 4C). Consistent with these findings, VSV replicated in the brains of the KI and *Ifit2−/*− mice to much higher titers than in Wt mice at 4 and 6 days post infection (Fig. 4D). A 10^5^-fold increase in viral titer was observed in KI and *Ifit2*−/− mice. These results indicate that while the *Ifit2* KI mutant was expressed strongly in the brains of the infected mice, it failed to protect against VSV infection and pathogenic consequences.

## DISCUSSION

VSV, a prototypic rhabdovirus, has a broad tissue tropism in mice. When intranasally inoculated, the virus directly transits to the olfactory bulb and spreads rapidly throughout the brain, with neurons being the primary target cells (24). When injected subcutaneously into footpads, the virus infects peripheral neurons and traffics to the brain via the spinal cord. The IFN-induced protein, IFIT2, protects mice from neuropathy in both experimental models as demonstrated by the high susceptibility of *Ifit2−/*− mice to VSV and other neurotropic RNA viruses (20, 21, 23, 26). Surprisingly, the observed *in vivo* antiviral action of *Ifit2* is quite tissue specific. In *Ifnar−/*− mice, peripheral VSV infection led to robust viral replication in many organs indicating that the type I IFN system is globally protective. In contrast, in *Ifit2−/*− mice, only the neurons of the CNS and PNS were susceptible to infection, as other tissues were well protected (20, 23). These results indicated that other ISGs protect different tissues, with IFIT2 dominantly protecting cells in the nervous system. As the biochemical mechanism of IFIT2’s antiviral action is not known, we used genetically modified mice to define the mode of antiviral action of murine IFIT2.

Using the newly generated conditional *Ifit2* knockout mice, we asked whether to provide protection, *Ifit2* needs to be induced in neuronal cells. In the intranasal infection model, our results clearly showed that *Ifit2* expression in neuronal cells is required for its protective action. Nes-Cre is expressed early in neuroectoderm differentiation (27) and, hence, in addition to neurons, astrocytes, and oligodendrocytes, would have lost the *Ifit2* gene in our conditional knockout mice. Nonetheless, we speculate that its expression in neurons, the site of VSV replication, was essential for its antiviral activity. In the future, to provide stronger evidence for the above conclusion, experiments that use mice expressing Cre-driven glial or other neuronal promoters will be required. In contrast to the intranasal model, in the footpad model, the absence of *Ifit2* in only neuronal cells did not make the mice as susceptible as *Ifit2−/*− mice. This observation indicates that *Ifit2* has anti-VSV activity in some cell types, other than neurons, which prevents entry of the virus to the nervous system. We have reported IFIT2’s protective effects against respiratory infection by Sendai virus (28), which presumably involved non-neuronal cells such as pulmonary epithelial cells or infiltrating immune cells. Further research will be needed to identify the cell type that provides the IFIT2-mediated protection from foot pad infection.

Identification of the biochemical basis of IFIT2’s action against VSV or other neurotropic RNA viruses has been challenging, primarily because of the absence of a suitable cell culture system that recapitulates the observations made in mice. Through their TPR motifs, the IFIT proteins can bind specific cellular proteins, such as components of the translation initiation factor 3 (29, 30), which, in turn, may affect viral protein synthesis. These proteins can also bind RNAs by recognizing A:U-rich sequences in the untranslated regions of mRNAs such as IFNB mRNA (31). It has been reported that in influenza virus-infected human cells, human IFIT2 binds to both cellular and viral mRNAs and facilitates their translation (32). In the current study, we asked whether the RNA-binding activity of IFIT2 was necessary for its anti-VSV activity *in vivo*. Mutations of two amino acids eliminated IFIT2’s RNA-binding ability *in vitro,* and expression of this mutant protein in mice was insufficient to confer antiviral effects against VSV. We anticipate that the RNA-binding defective mutant of IFIT2 is ineffective against other neurotropic RNA viruses that are susceptible to the antiviral action of IFIT2. Our observations suggest that for its antiviral action in the brain, IFIT2 requires to bind to RNAs possessing specific sequences or structural motifs. Because such structural features are unlikely to be present in the genomes of many neurotropic viruses that are inhibited by IFIT2, the candidate RNAs are likely to be cellular in origin. Moreover, their expression is expected to be restricted to neurons because of the observed tissue specificity of IFIT2 action. Future analysis of cells from the KI mouse should reveal the identities of the cellular RNAs that bind IFIT2 in the brains of the infected mice and the functional consequences of this binding.

## MATERIALS AND METHODS

### Mice

All mice were of C57BL/6 background, 8 to 16 weeks old, and of both sexes. *Ifit2−/*− mice were previously described (20). Floxed Ifit2 mice (*Ifit2 fl/fl*), carrying loxP-flanked Ifit2 exon 3 alleles, with no remaining neomycin resistance gene, were custom generated by Taconic/Xenogen. *Ifit2 fl/fl* mice were crossed with Cre recombinase-expressing mice to conditionally delete *Ifit2* either ubiquitously (CMV-promoter Cre mice, The Jackson Laboratory 6054) or in cells of the central nervous system (Nestin-promoter Nes-Cre mice, The Jackson Laboratory 3771). The presence of the floxed and the deleted *Ifit2* alleles in mouse genomic DNA was detected by PCR using primers CCF24NeodelF (5′-GGCAGGAACCAATCTGAGACTACGG-3′), CCF24NeodelR (5′-AAGACCGAAGTCATTTCTGTGTCCCTT-3′) and CCF24_CKO_delF (5′-CTCTCCCAGGGATGAGCTATCTAATTG-3′). The *Ifit2* KI mouse expresses a mutant IFIT2, which cannot bind RNA. The KI mouse was custom generated by replacing, in the Ift2 resident gene, the codons for R287 and K406 with codons for glutamic acid. Genome sequencing confirmed the presence of only the desired mutations in the *Ifit2* gene of the KI mouse. C57BL/6 wild-type mice were obtained from Taconic Farms.

### Virus infections and pathogenesis

VSV Indiana was a gift from Amiya K. Banerjee, Lerner Research Institute, Cleveland, Ohio. For intranasal infections, 4 × 10^2^ pfu of VSV in 35 µL of endotoxin-free phosphate-buffered saline (PBS) was inhaled by isoflurane-anesthetized mice of either sex, with PBS-only as control. For subcutaneous infection, 10^6^ pfu of VSV was injected into the left hind leg footpad of anesthetized mice (22). Thereafter, mice were monitored daily for weight loss and development of neurological symptoms. The appearance of hind leg paralysis was the hallmark of disease for mice infected with VSV subcutaneously. For mice receiving intranasal infection, neurological symptoms, such as ataxia, hind limb paralysis, and hyper-excitability, preceded death.

### Primary splenocytes

Induction of Ifit2 protein in Ifit2 fl/fl mice was tested using primary Wt and Ifit2 fl/fl splenocytes. Spleens were harvested and mashed through a 70-µm sieve with a syringe plunger. Cells were washed in PBS, and after lysis of red blood cells with ammonium chloride/EDTA buffer and washing with PBS, cells were seeded into 10% fetal bovine serum/Dulbecco's modified Eagle’s medium and immediately interferon treated overnight for immunoblot.

### Immunoblot

Primary splenocytes were stimulated with 1,000 U/mL of murine IFN-β (PBL Assay Science) and lysed in alkaline lysis buffer (50 mM Tris [pH 7.6], 150 mM NaCl, 0.2% Triton X-100, 1 mM sodium orthovanadate, 10 mM sodium fluoride, 5 mM sodium pyrophosphate, 10 mM β-glycerophosphate, and complete EDTA-free protease inhibitor [Roche]). Cell extracts were separated via 10% SDS-PAGE, transferred to polyvinylidene difluoride membranes, blocked with 5% dry milk in Tris-buffered saline/0.05% Tween- 20 overnight, and labeled with anti- mouse IFIT3/P49, anti-mouse IFIT2/P54, anti-mouse IFIT1/P56 polyclonal rabbit sera (raised in our lab, 15), or anti-β-actin mouse monoclonal antibody (Sigma, clone AC-15) as control.

### Virus titration by plaque assay

For quantification of infectious VSV in organs, mice were anesthetized with pentobarbital; blood was removed from organs by cardiac perfusion with 10 mL of PBS. Organs were snap-frozen in liquid nitrogen, weighed, and pestle/tube homogenized (Kimble/Kontes) in PBS followed by pelleting of debris by centrifugation at 10,000 × *g* for 15 min. Virus titers were determined in 10-fold serial dilutions on Vero cells by plaque assay. Results are expressed as pfu per gram of tissue.

### Purification of recombinant IFIT2 proteins

Murine Ifit2 cDNA was cloned into pGEX-6P-3 (GE Healthcare) to be bacterially expressed with an N-terminal GST-tag. Ifit2-RK (R287E, K406E) was designed based on published human IFIT2 RNA-binding data (25) using the Q5 site-directed mutagenesis kit (NEB). *E. coli* BL21(DE3) transformants were grown to optical density of 0.7 in 500 mL of Luria-Bertani broth/ampicillin, and GST fusion protein expression was induced by 0.25 mM isopropyl β-D-1-thiogalactopyranoside at 16°C for 16 h. Bacteria were lysed in 20 mL of high salt buffer to dissociate bacterial RNA (1 M NaCl, 20 mM Tris [pH 7.5], 10% glycerol, 0.2% NP-40, 1 mM dithiothreitol) aided by lysozyme and sonication after freezing once. GST proteins were purified using 1 mL of GST Sepharose 4B slurry (GE Healthcare) and 10 mM reduced glutathione in 50 mM Tris (pH 8) for elution, followed by dialysis in dialysis buffer (50% glycerol, 150 mM NaCl, 50 mM Tris [pH 7.5]).

### Electrophoretic mobility shift assay

Single-stranded 32-nt AU-rich RNA (5′-AUUAAUUUAUAAUUUAAAUUAUUUUCUACUUU-3′; IDT DNA) was end labeled by T4 phosphonucleotide kinase (Promega) using radioactive [γ-^32^P]ATP (3,000 Ci/mmol, Perkin-Elmer) for 1 h at 37°C and purified by phenol/chloroform and Sephadex G-25 (Quick Spin Oligo Columns, Roche). Ten femtomoles of labeled RNA (~100 pg) was mixed with buffer or 5 ng (50× excess) of competitor RNAs (poly-uridylic acid, poly-guanylic acid, poly-adenylic acid, poly-cytidylic acid, or poly-adenylic:uridylic acid; Santa Cruz Biotechnology) in a final reaction volume of 10 µL (in 9% glycerol, 50 mM Tris [pH 8], 90 mM NaCl, 1 mM MgCl_2_, 0.05% Triton X-100). This included 250 nM GST, GST-Ifit2, or GST-Ifit2-RK proteins or buffer. Binding occurred during 30 min on ice, after which the reaction products were separated by 6% native polyacrylamide gel electrophoresis (National Diagnostics) with 0.5× tank buffer at 4°C, followed by exposure of the dried gel to film.

### Statistical analysis

Where appropriate, *t*-tests were conducted to confirm significance of difference between two groups, using an unpaired two-tailed *t*-test with a 95% confidence interval, employing Prism 5.02 software (GraphPad). For survivals, a log-rank test (Mantel–Cox) was used to determine the significance of differences. *P* values are represented in the figure legends by asterisks: *P ≤ 0.05, **P ≤ 0.01, ***P ≤ 0.001; for *P* values larger than 0.05, the differences were considered not significant. All experiments were performed at least twice independently.
